# Cathepsin V ( *CTSV* ) Shapes the Epigenetic–Immune Landscape of Bladder Cancer and Emerges as a Noninvasive Biomarker

**DOI:** 10.1111/iju.70609

**Published:** 2026-08-26

**Authors:** Wallax Augusto Silva Ferreira, Fabio Albuquerque Marchi, Glauco Akelinghton Freire Vitiello, Rolando André Rios Villacis, Ana Paula Drummond Rodrigues, Edivaldo Herculano Correa de Oliveira

**Affiliations:** ^1^ Laboratory of Cytogenomics and Environmental Mutagenesis, Environment Section (SEAMB) Evandro Chagas Institute (IEC) Ananindeua PA Brazil; ^2^ Department of Head and Neck Surgery University of São Paulo Medical School São Paulo SP Brazil; ^3^ Center for Translational Research in Oncology Cancer Institute of the State of São Paulo (ICESP) São Paulo SP Brazil; ^4^ Laboratory of DNA Polymorphisms and Immunology, Department of Pathological Sciences, Biological Sciences Center Londrina State University (UEL) Londrina PR Brazil; ^5^ Department of Genetics and Morphology, Institute of Biological Sciences University of Brasília (UnB) Brasília DF Brazil; ^6^ Multiuser Cellular Biology and Ultrastructure Laboratory, Section of Hepatology Evandro Chagas Institute (IEC) Belém PA Brazil; ^7^ Institute of Exact and Natural Sciences, Faculty of Natural Sciences Federal University of Pará (UFPA) Belém PA Brazil

**Keywords:** biomarker, cathepsin V, *CTSV*, cysteine, DNA methylation, epigenetic, tumor microenvironment

## Abstract

**Objective:**

The aim of this study was to perform a comprehensive characterization of *CTSV* regulation in bladder cancer (BC) and its association with clinicopathological features, prognosis, and tumor stroma modulation using multi‐omics databases.

**Methods:**

We used integrative bioinformatics approaches with publicly available multi‐omics datasets. *CTSV* expression was evaluated across BC cohorts using TIMER2.0, GEPIA, GENT2, and GEO databases, while protein expression was examined with the Human Protein Atlas. Genomic alterations, DNA methylation, and histone modifications were analyzed using TCGA, UALCAN, ASD MethSurv, SMART, and DepMap databases. Associations with clinical outcomes, immune infiltration, and drug sensitivity were investigated through TCGA, CIBERSORT, TISIDB, single‐cell and spatial transcriptomics, and RNAactDrug platforms.

**Results:**

We found that *CTSV* was upregulated in BC, especially in non‐papillary tumors. Moreover, its expression correlated positively with tumor grade, the presence of distant and lymph node metastases, resistance to therapy, and poorer overall and progression‐free survival. Furthermore, *CTSV* expression was associated with mutations in known BC driver genes (*TP53*, *RB1*, and *ERBB2*) and showed a correlation with promoter hypomethylation, gene body methylation, and histone markers (e.g., H3K18ac1K23ac0), suggesting an epigenetic mechanism driving *CTSV* expression. The *CTSV* ^*high*^ and *CTSV* ^*low*^ groups exhibited a different stromal composition, with the former showing a more immunogenic signature. Finally, we found that cell lines and tumors highly expressing *CTSV* exhibit higher sensitivity to multiple chemotherapeutic drugs.

**Conclusion:**

*CTSV* overexpression is associated with bladder cancer aggressiveness through epigenetic regulation and immune modulation, emerging as a promising prognostic biomarker and noninvasive diagnostic target.

## 1 Introduction

Bladder cancer (BC) is the most common malignant tumor of the urinary tract and occurs more frequently in men in Western Europe and North America. Major risk factors include smoking, family history, diet, and occupational or environmental exposures. Based on muscle invasion, BC is classified as muscle‐invasive bladder cancer (MIBC) and non‐muscle‐invasive bladder cancer (NMIBC), with approximately 75% of patients diagnosed with NMIBC. For high‐grade cases and/or early subepithelial invasion, intravesical Bacillus Calmette–Guérin therapy is often used. However, recurrence affects more than 50% of NMIBC patients, and 15%–40% progress to MIBC or metastasis, leading to a poor five‐year survival rate. The standard treatment for MIBC is radical cystectomy, while metastatic disease is managed with chemotherapy or immune checkpoint inhibitors (ICIs), though response rates remain limited [1].

Bioinformatic multi‐omics analyses have revolutionized our understanding of the molecular landscape of BC, identifying distinct molecular subtypes and supporting precision medicine strategies [2, 3]. However, clinical translation faces challenges, including nonstandardized classifications, technical complexity, costs, and limited validation [4]. Therefore, sensitive and specific biomarkers are urgently needed for early detection and improved management.

Cathepsin V (*CTSV*), also known as *CATL2*, *CTSL2*, or *CTSU*, is a human cysteine protease primarily localized in endolysosomes. It regulates extracellular matrix (ECM) remodeling, antigen processing, neuropeptide cleavage, angiogenesis, vascular homeostasis, epidermal differentiation, and thyroid hormone regulation [4]. Dysregulated *CTSV* contributes to tumorigenesis and cancer progression. However, its role in BC remains poorly understood. Here, we investigated *CTSV* expression in BC using multi‐omics analyses and assessed its epigenetic modulation, prognostic value, and correlation with tumor‐infiltrating immune cells.

## 2 Material and Methods

### 2.1 Data Collection

To explore *CTSV* expression across cancers, we analyzed TCGA datasets using TIMER2.0 [5] and the GEPIA databases [6], which include normal samples from GTEx. Pan‐cancer validation was performed with the GENT2 database. To further validate the differential expression of *CTSV* found in TCGA‐BLCA (404 tumor and 28 normal samples), we used the GSE7476 dataset (185 tumor and 59 normal samples) [7]. Additionally, *CTSV* expression was examined in 1773 human cancer cell lines, including 38 bc cell lines, through the DepMap database [8].

To investigate *CTSV* expression in extracellular vesicles (EVs), we used the exoRBase 2.0 database (http://www.exorbase.org/exoRBaseV2/toIndex), which aggregates RNA‐seq data from 905 biofluid‐derived samples. The expression values were log2‐transformed for subsequent analyses.

### 2.2 Human Protein Atlas (HPA) Database Analysis

Confocal microscopy images of *CTSV* in A‐431 and Hep G2 cell lines, as well as immunohistochemistry images of normal and tumor tissues, were retrieved from the Human Protein Atlas. All samples were analyzed with the CAB017112 antibody to ensure consistency.

### 2.3 Clinical Correlations and Survival Analysis

Normalized RNA‐seq data from TCGA‐BLCA were obtained via the R2 platform, with corresponding clinical data from the Genomic Data Commons after excluding incomplete records. Validation was performed using the GSE32894, GSE31684, GSE87304, and GSE169455 cohorts. Survival analyses of relapse‐free and overall survival in TCGA‐BLCA were conducted using the Kaplan–Meier plotter and Human Protein Atlas, while overall survival was further validated in GSE13507 and GSE48276 through PROGgeneV2. Patients were classified into high‐ or low‐expression groups based on the median *CTSV* expression.

### 2.4 Genomic and Epigenetic Alterations

*CTSV* mutation profiles and copy number variations (CNVs) were characterized with cBioPortal, and correlations with expression were assessed using Spearman tests. The clinical relevance of CNV‐related alterations was evaluated using GSCA, while the impact of 20 key driver gene mutations on *CTSV* expression was examined with CAMOIP. Epigenetic regulation was assessed through UALCAN, which provided promoter methylation data from TCGA‐BLCA. Single‐CpG methylation patterns were further analyzed using MethSurv, based on Illumina HM450K array data. Probe‐level differential methylation and correlations with mRNA levels were calculated using SMART. Histone modification data for bladder cancer cell lines were obtained from DepMap via the IMOPAC database, while cell‐free DNA methylation patterns were explored using bisulfite sequencing data from GSE79279, processed through the cfOmics database.

### 2.5 Immune Infiltration Analysis

The relationship between *CTSV* expression and immune features was investigated using TISIDB, which categorizes TCGA‐BLCA tumors into immune subtypes and provides signatures for 28 immune cell types. Immune cell abundance was estimated using gene set variation analysis, and the CIBERSORT algorithm quantified 22 immune populations within *CTSV* ^*high*^ and *CTSV* ^*low*^ groups.

### 2.6 Single‐Cell and Spatial Transcriptomics

Single‐cell RNA‐seq data from bladder tumors were retrieved from the TISCH2 database [9], including GSE130001 (4129 cells from two patients) and GSE149652 (15 538 cells from seven patients treated with chemo‐ or immunotherapy). Spatial expression patterns and colocalization with immune markers were assessed using the SORC database, specifically leveraging samples GSM5224027 and GSM5224029 from the GSE171351 dataset, which contains treatment‐naïve muscle‐invasive bladder cancers.

### 2.7 Drug Sensitivity

Associations between *CTSV* expression and drug responses were analyzed using RNAactDrug with stringent filtering (FDR = 0.01, *p* < 0.01, CCLE source). The six most strongly correlated compounds were identified by Spearman analysis. In parallel, drug sensitivity data from TCGA‐BLCA were analyzed with the CPADS tool, stratifying patients by *CTSV* expression.

### 2.8 Statistical Analysis

Cox regression models were used to identify independent prognostic factors for overall survival in TCGA‐BLCA, with hazard ratios and 95% confidence intervals reported. Kaplan–Meier curves and log‐rank tests compared survival between expression groups. Logistic regression examined associations between *CTSV* expression and clinicopathological parameters. Receiver operating characteristic curves and area under the curve values were calculated to assess diagnostic accuracy, with cutoffs defined by the Youden index. Analyses were considered significant at *p* < 0.05, and visualizations were generated using the R package ggplot2 (v3.3.6).

## 3 Results

### 3.1 *CTSV* Is Upregulated in Multiple Cancer Types

**FIGURE 1:**
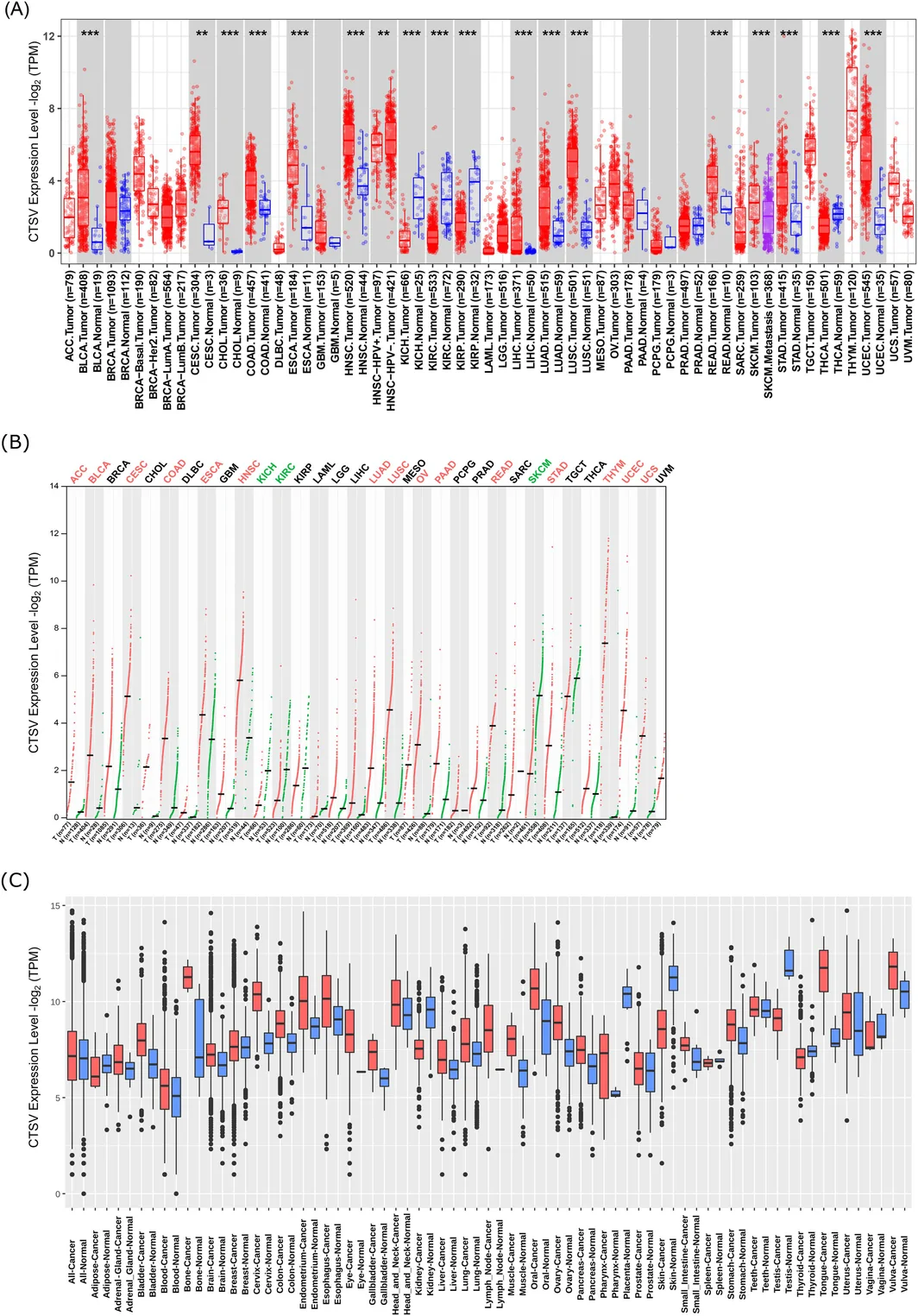
Differential expression profile of *CTSV* across human cancers. (A) *CTSV* expression in different tumors from the TCGA cohort. Red box plot: Tumor tissue. Blue box plot: Adjacent nontumor tissues. (B) Differential *CTSV* expression in various malignancies (TCGA) and normal samples (GTEx) using GEPIA2 database. Red: Tumor samples. Green: Normal samples. (C) Tissue‐wide gene expression pattern of *CTSV* across 72 paired tissues obtained from the GENT2 database. Red: Cancer samples. Blue: Normal samples. TPM: Transcripts per million. *p < 0.05, **p < 0.01, ***p < 0.001.

We first evaluated *CTSV* expression across multiple cancer types using TCGA unpaired RNA‐seq data. *CTSV* was overexpressed in a wide range of human tumors (BLCA, CESC, CHOL, COAD, ESCA, HNSC, LIHC, LUAD, READ, SKCM, STAD, and UCEC) (Figure 1A). Given the limited number of normal samples in TCGA, we expanded our analysis by including normal samples from the GTEx database, which confirmed our initial results (Figure 1B). In addition, *CTSV* dysregulation was validated in the GPL570 cohort (Figure 1C), confirming it as a widespread oncogenic event.

### 3.2 *CTSV* Is Overexpressed in BC Tissues, Cell Lines and Exosomes

**FIGURE 2:**
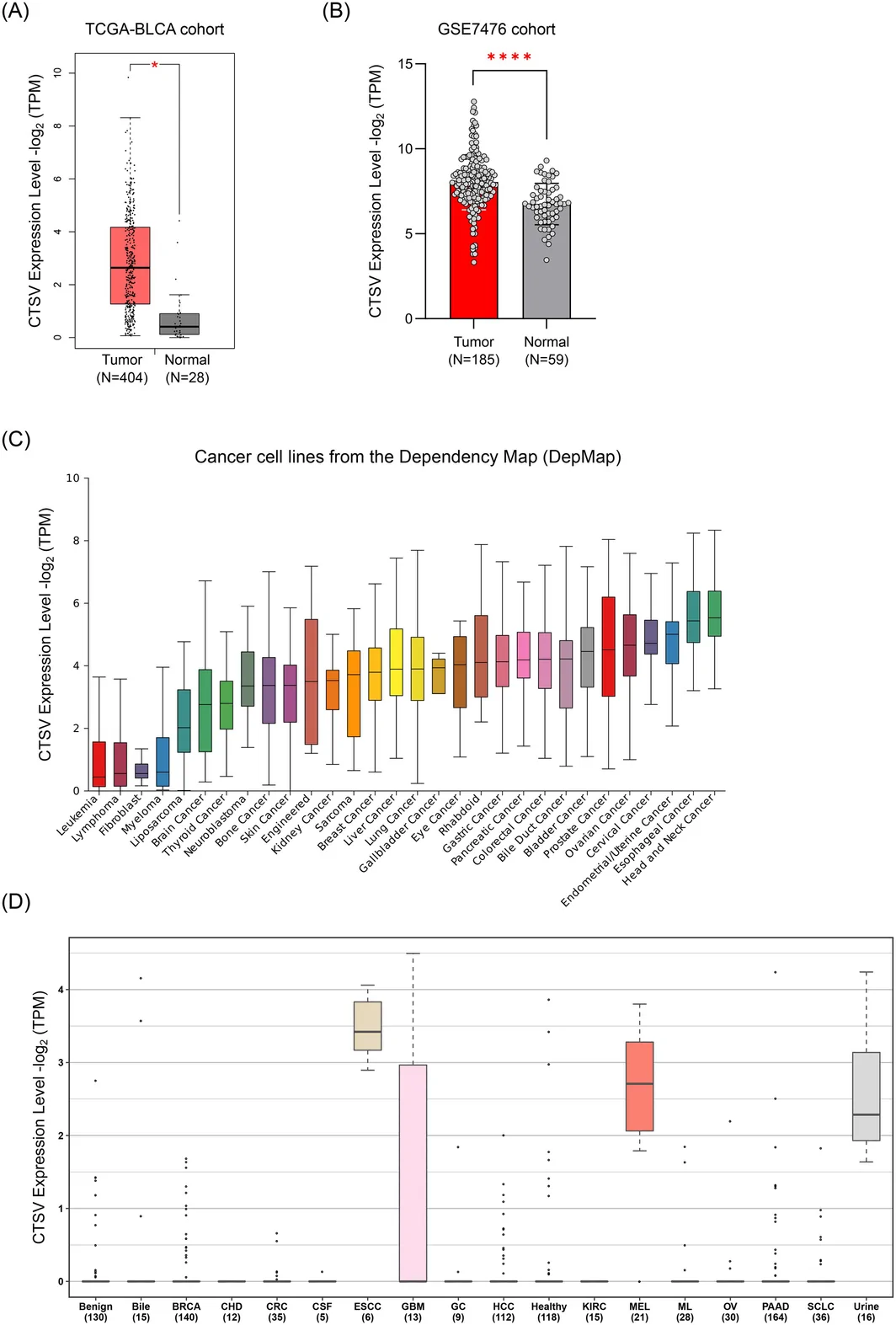
*CTSV* expression is increased in BC. (A) *CTSV* mRNA expression was higher in BC tissues than in paired non‐tumor tissues in TCGA‐BLCA cohort (BC = 404 samples; Normal = 28 samples). (B) Similar results were observed in the GSE7476 cohort (BC = 185 samples; normal = 59 samples). (C) *CTSV* expression across multiple human cancer cell lines from DepMap database, including BC cell lines. (D) Expression profiles of *CTSV* in extracellular vesicles (EVs) derived from human biofluids under various biological conditions, including healthy individuals, benign disease, and cancers such as breast cancer (BRCA), coronary heart disease (CHD), colorectal cancer (CRC), cerebrospinal fluid (CSF), esophageal squamous cell carcinoma (ESCC), glioblastoma (GBM), gastric cancer (GC), hepatocellular carcinoma (HCC), kidney cancer (KIRC), melanoma (MEL), malignant lymphoma (ML), ovarian cancer (OV), pancreatic adenocarcinoma (PAAD), and small cell lung cancer (SCLC).

We next focused on the differential expression of *CTSV* in BC. *CTSV* mRNA was consistently upregulated in BC tumors compared with normal tissues in the TCGA‐BLCA and GSE7476 cohorts (Figure 2A,B), and was highly expressed in BC cell lines (Figure 2C). Importantly, *CTSV* was detectable in exosomes from urine and from other cancers (esophageal carcinoma, GBM, and melanoma) (Figure 2D), suggesting its potential as a liquid biopsy biomarker.

**FIGURE 3:**
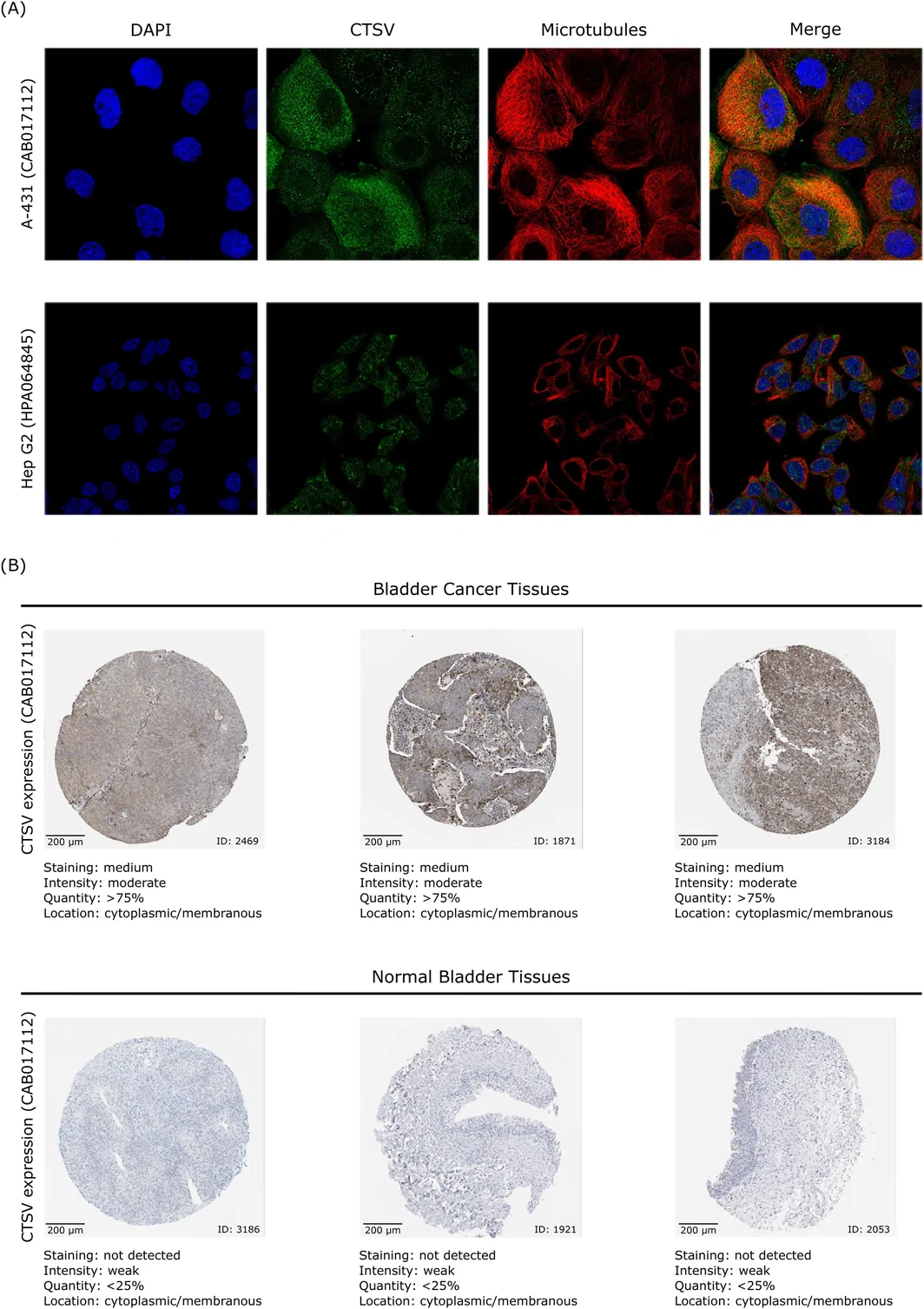
CTSV protein expression analysis. (A) Subcellular localization of CTSV (green) in the cytosol and nucleus of A‐431 and Hep G2 cell lines. Scale: 10 μm. (B) Representative immunohistochemistry of CTSV protein in BC tissues (upper panel; patient IDs: 2469, 1871, and 3184) and normal tissues (lower panel; patient IDs: 3186, 1921, and 2053). Scale bars: 200 μm. All samples were stained using the CAB017112 antibody.

Subcellular analysis showed that *CTSV* was localized in the cytosol (as punctate lysosomal staining) and the nuclear compartment (nucleolar fibrillar center) in A‐431 and Hep G2 cells (Figure 3A). In addition, immunohistochemistry (IHC) confirmed high *CTSV* levels in BC tissues compared to normal counterparts (Figure 3B), supporting our mRNA results.

### 3.3 Clinicopathological Features Impact *CTSV* Expression

**FIGURE 4:**
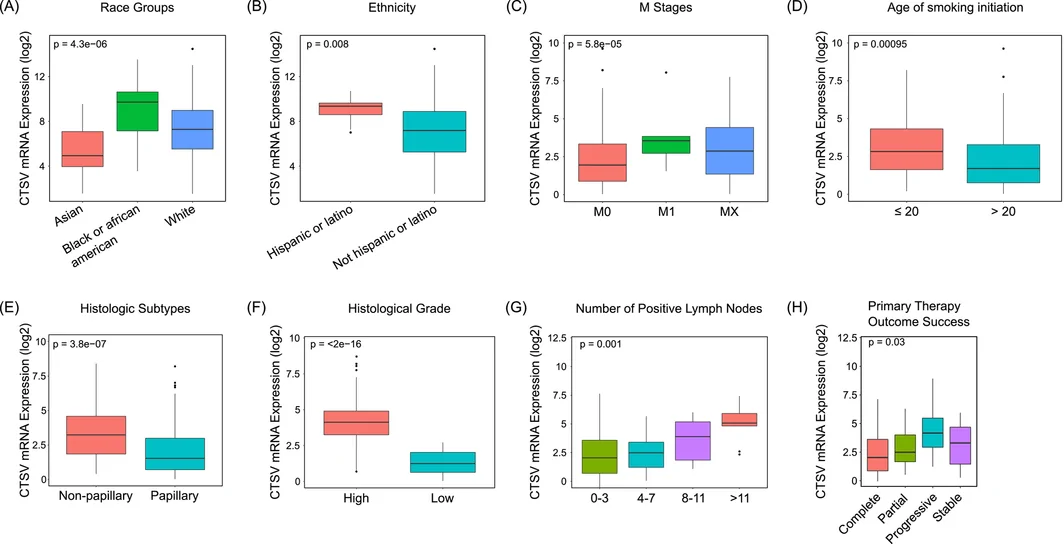
Cathepsin V (*CTSV*) levels correlate with BC characteristics. (A) *CTSV* expression across race groups in the TCGA‐BLCA dataset. *p* < 0.001 by Kruskal–Wallis test. (B) *CTSV* expression by ethnicity in the TCGA‐BLCA dataset. (C) *CTSV* expression across different M stages (*p* = 5.8e‐05 by Kruskal–Wallis test). (D) *CTSV* expression at different ages of smoking initiation. (E) *CTSV* expression across different histologic subtypes (*p* = 3.8e‐07 by Wilcoxon test). (F) *CTSV* expression in different histologic grades (*p* < 2e‐16 by Wilcoxon test). (G) *CTSV* expression according to the number of positive lymph nodes (hematoxylin and eosin stain—HE) (*p* = 0.001 by Kruskal–Wallis test). (H) *CTSV* expression according to primary therapy outcome success: Complete response, partial response, progressive disease, and stable disease in TCGA‐BLCA cohort (*p* = 0.03 by Kruskal–Wallis test).

*CTSV* expression was significantly higher in patients from Black/African American and Hispanic/Latin populations, as well as in early‐onset smokers. Additionally, *CTSV* was enriched in non‐papillary tumors, high‐grade tumors, and those with advanced M stage, and increased progressively with lymph node positivity. Patients with progressive disease had higher *CTSV* expression, whereas responders had lower expression (Figure 4; Table S1).

Validation in multiple cohorts (GSE32894, GSE31684, GSE87304, and GSE169455) confirmed that *CTSV* expression is associated with molecular and LundTax subtypes, as well as chemotherapy responsiveness (Table S1). Logistic regression further showed that *CTSV* expression was significantly associated with tumor histology (OR = 0.3107, *p* = 0.0001), ethnicity (OR = 7.198, *p* = 0.0416), lymphovascular invasion (OR = 0.6218, *p* = 0.0449), histologic grade (OR = 11.82, *p* = 0.0001), and sample type (OR = 9.147, *p* = 0.0033) (Table 1; Table S1), highlighting its potential diagnostic value in BC.

**TABLE 1:** *CTSV* expression is associated with clinical‐pathological characteristics in the TCGA‐BLCA cohort (logistic regression).

Characteristics	Odds ratio (OR)	95% CI	*p*
Tumor histology			
Papillary vs. non‐papillary	0.3107	0.2005–0.4753	0.0001
Ethnicity			
Not hispanic or latino vs. hispanic or latino	7.198	1.263–135.2	0.0416
Lymphovascular invasion			
Yes vs. no	0.6218	0.3898–0.9876	0.0449
Histologic grade			
High grade vs. low grade	11.82	6.997–20.50	0.0001
Sample type			
Tumor vs. normal	9.147	2.578–58.14	0.0033

### 3.4 Prognostic and Diagnostic Significance of *CTSV* for BC Patients

**FIGURE 5:**
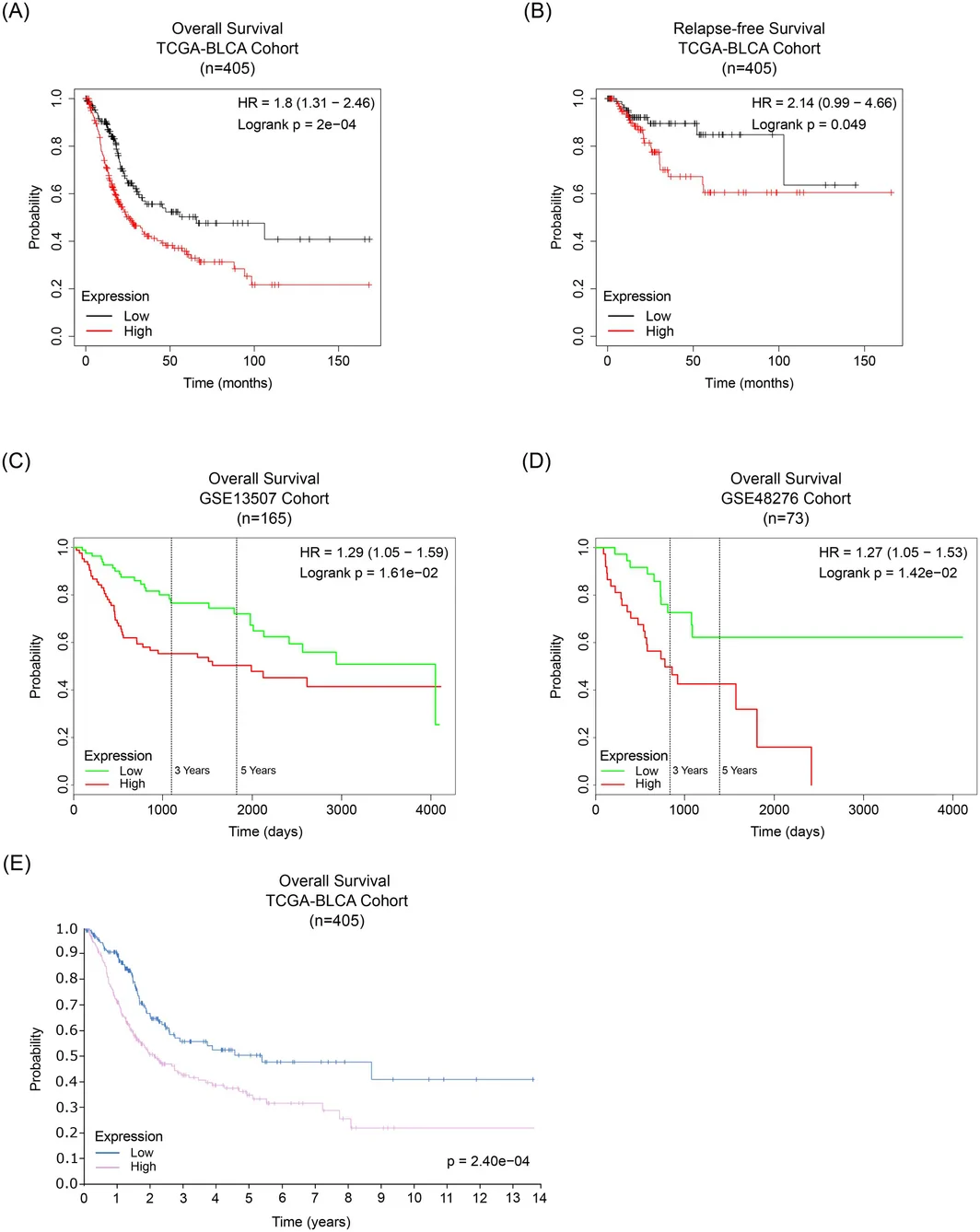
High *CTSV* expression is associated with poor prognosis. (A, B) Kaplan–Meier survival curves for overall survival (OS) and relapse‐free survival (RFS) in the TCGA‐BLCA cohort. (C, D) Overall survival (OS) curves for *CTSV* expression in the GSE13507 and GSE48276 cohorts. (E) Patients with low *CTSV* protein levels had prolonged overall survival (OS) in the TCGA‐BLCA cohort (*n* = 405; *p* = 0.00024). Red and black lines indicated above‐ and below‐median expressions, respectively. HR, hazard ratio.

**FIGURE 6:**
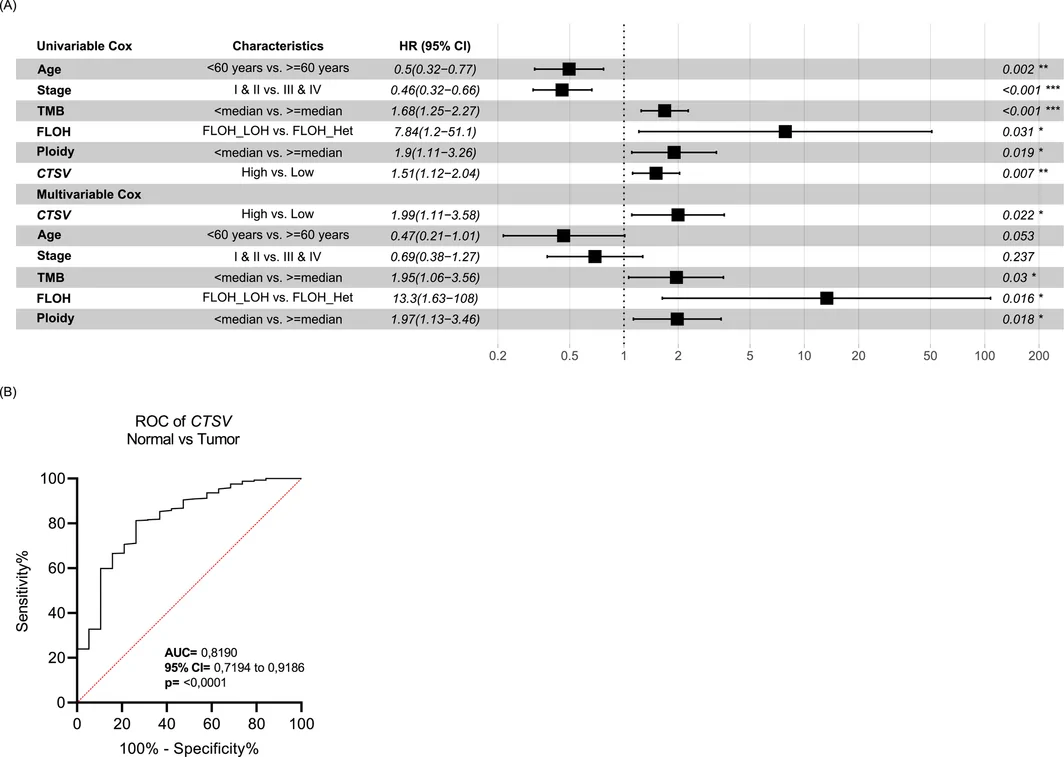
(A) Univariate and multivariate independent prognostic analyses of *CTSV* expression and clinical data in the TCGA‐BLCA cohort. TMB, tumor mutational burden; FLOH, focal loss of heterozygosity. (B) ROC curve illustrating the diagnostic performance of *CTSV* expression in distinguishing BLCA from non‐tumor tissue in the TCGA‐BLCA cohort.

Survival analyses showed that high *CTSV* expression predicts poor overall survival (OS) and recurrence‐free survival (RFS) (Figure 5A–D). Similarly, high *CTSV* protein levels also correlated with poor outcomes (Figure 5E). Cox regression (univariate HR = 1.51; multivariate HR = 1.99, *p* = 0.022) confirmed *CTSV* as an independent OS predictor (Figure 6A).

ROC analysis demonstrated that *CTSV* mRNA effectively distinguishes BC from normal tissues (AUC = 0.8190, *p* < 0.001) (Figure 6B). These results highlight *CTSV* as both a prognostic and diagnostic biomarker.

### 3.5 *CTSV* Expression Is Associated With Copy Number Alterations (CNAs) and Mutations in Driver Genes

**FIGURE 7:**
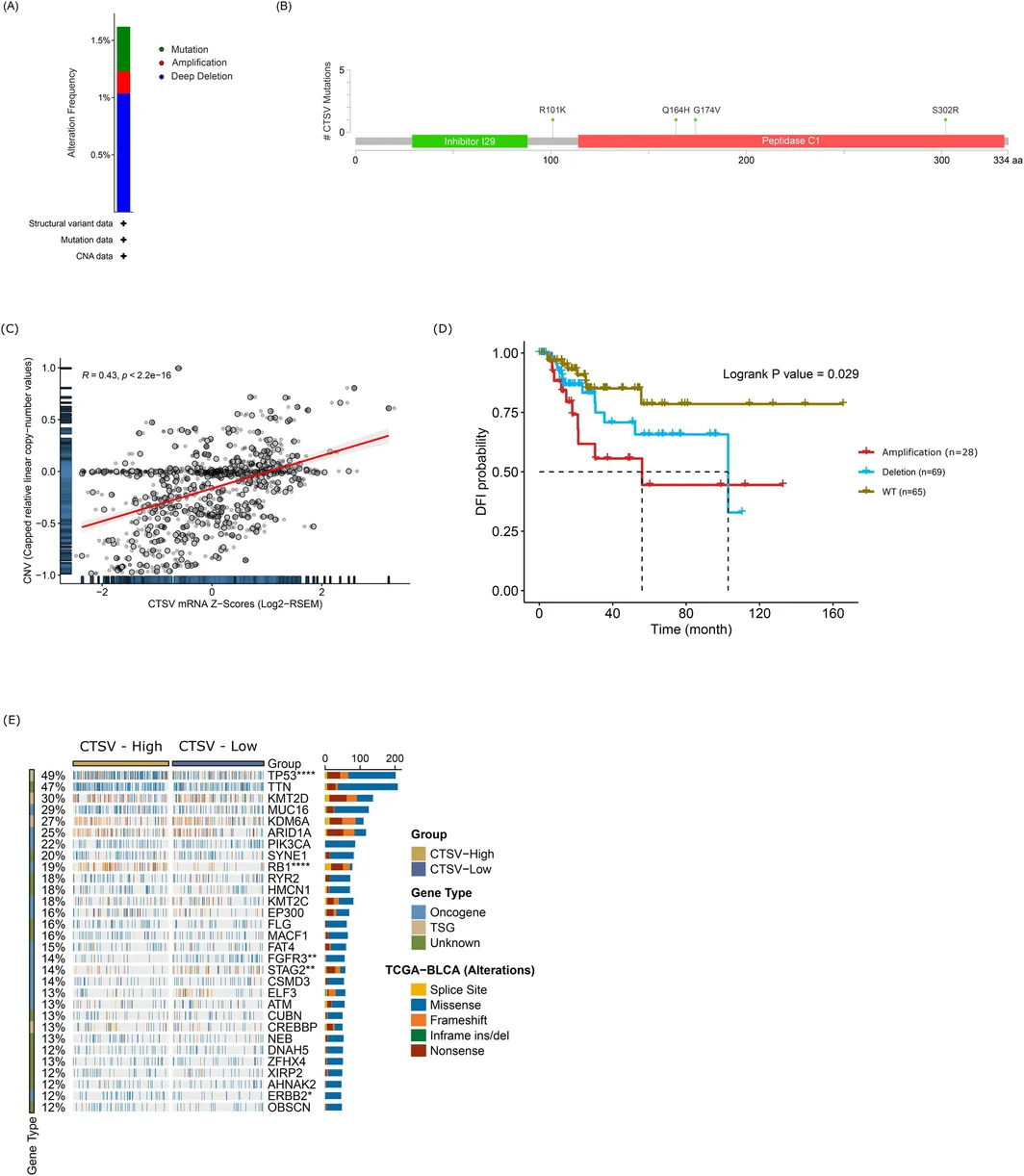
*CTSV* alteration profile across different BC cohorts. (A) Analysis of *CTSV* gene alterations, including mutations (green), amplification (red), and deep deletions (blue). (B) Distribution of mutations along the *CTSV* domains in BC. (C) *CTSV* expression levels are strongly correlated with copy number variation (CNV). (D) Kaplan–Meier survival curves showing the DFI (disease‐free survival) difference between patients stratified by *CTSV* CNV groups (Red: *CTSV*‐ amplified; Blue: *CTSV*‐deleted; Green: *CTSV*‐wild‐type). (E) Mutational landscape of the top 30 cancer driver genes in TCGA‐BLCA patients exhibiting abnormal *CTSV* expression. BC patients were divided into high‐ and low‐groups based on median *CTSV* expression. Wicoxon test was conducted, and results with adjusted *p* < 0.05 are displayed. * *p* < 0.05; **p < 0.01; ***p < 0.001; ****p < 0.0001.

cBioPortal analysis revealed low mutation rates in *CTSV* across 1544 bc samples (1.62%). Most alterations were deep deletions (16 cases), followed by missense mutations (6 cases) clustered in the peptidase C1 domain (G174V, S302R, Q164H) (Figure 7A,B). *CTSV* expression positively correlated with CNV burden (ρ = 0.43, *p* = 2.2e‐16) and amplification predicted worse disease‐free interval (*p* = 0.029) (Figure 7C,D).

Driver gene analysis revealed *CTSV* ^*high*^ tumors harbored more *TP53*, *RB1*, and *ERBB2* mutations, while *CTSV* ^*low*^ tumors showed *FGFR3* and *STAG2* alterations (Figure 7E). These associations point to potential interactions between *CTSV* dysregulation and cancer driver gene activity.

### 3.6 *CTSV* Overexpression Is Strongly Associated With Epigenetic Changes in BC

**FIGURE 8:**
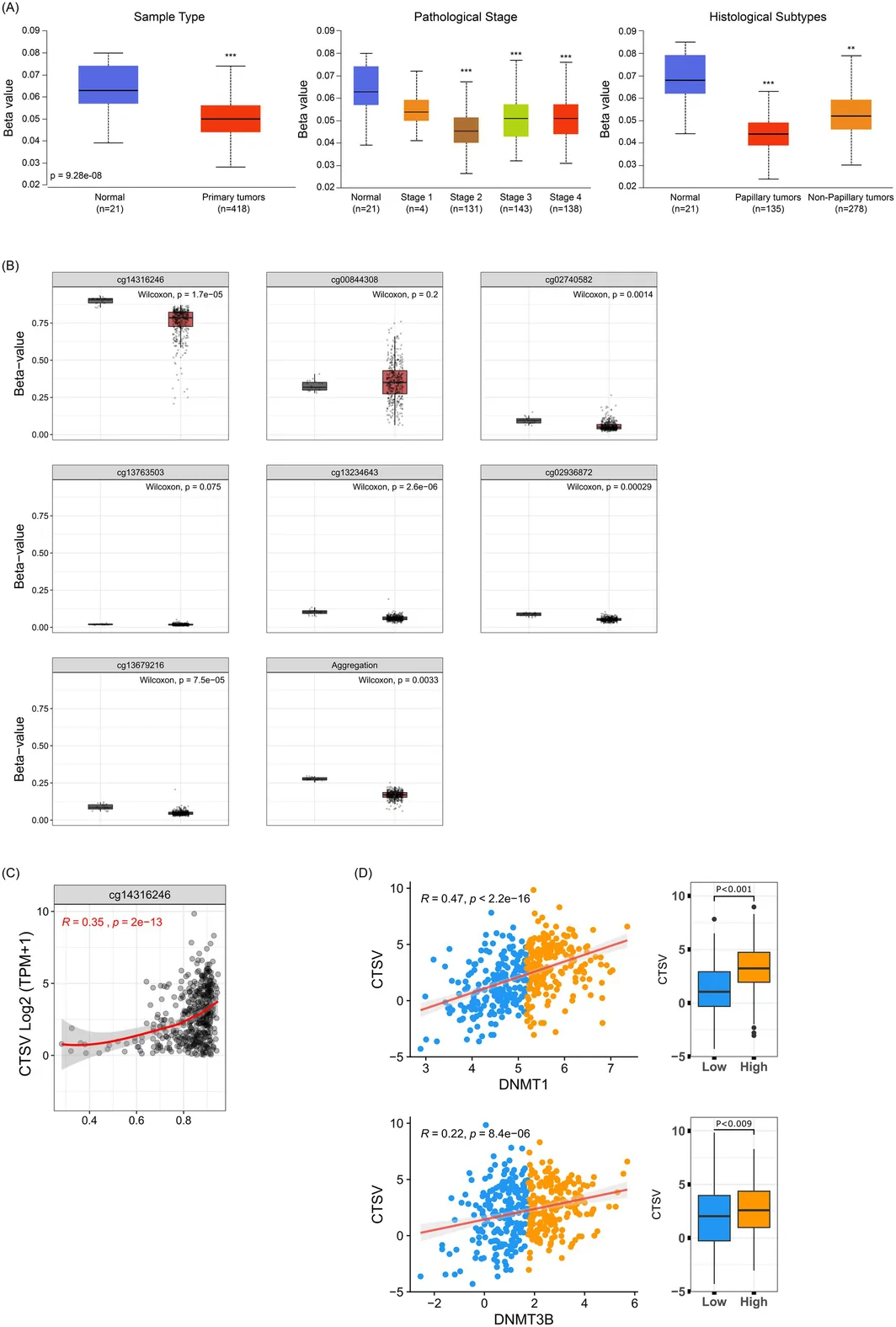
Hypomethylation of *CTSV* leads to upregulation in BC. (A) Comparison of *CTSV* methylation levels between normal subjects and BC patients. Box plots showing *CTSV* promoter methylation according to sample type (normal vs. tumor), clinical stage (I‐IV), and histological subtypes (normal, papillary, non‐papillary). (B) Differential methylation levels of seven *CTSV*‐associated CpG probes (cg14316246, cg00844308, cg02740582, cg13763503, cg13234643, cg02936872, and cg13679216) comparing BC samples (*N* = 424; red boxes) and normal samples (*N* = 28; gray boxes) from the TCGA database. (C) Spearman's correlation between methylation levels (β‐values, 450 K array) of the cg14316246 probe (located in gene body) and *CTSV* mRNA expression (log2‐scaled TPM + 1) in BC samples from TCGA. (D) Spearman's correlation between *CTSV* and DNA methyltransferases *DNMT1* and *DNMT3B*.

DNA methylation analysis revealed reduced *CTSV* promoter methylation in BC compared with normal patients (*p* = 9.28e‐08), a trend also observed in BC cell lines (Figure S1). CpG site analysis identified hypomethylation in five of seven *CTSV* probes, with cg14316246 (located in the gene body) positively associated with expression (*R* = 0.35, *p* = 2e‐13) (Figure 8B,C), fitting to a prominent role of gene body methylation (gbM) in regulating *CTSV* expression (Figure S2). There is compelling evidence for a causal relationship between gene body methylation (gbM) and stabilization of transcription and enhancement of oncogene expression [10, 11]. Moreover, *CTSV* expression was associated with the methylation‐related enzymes *DNMT1* (ρ = 0.47, *p* < 2.2e−16) and *DNMT3B* (ρ = 0.22, *p* = 8.4e−06) (Figure 8D), supporting the role of epigenetic regulation.

**FIGURE 9:**
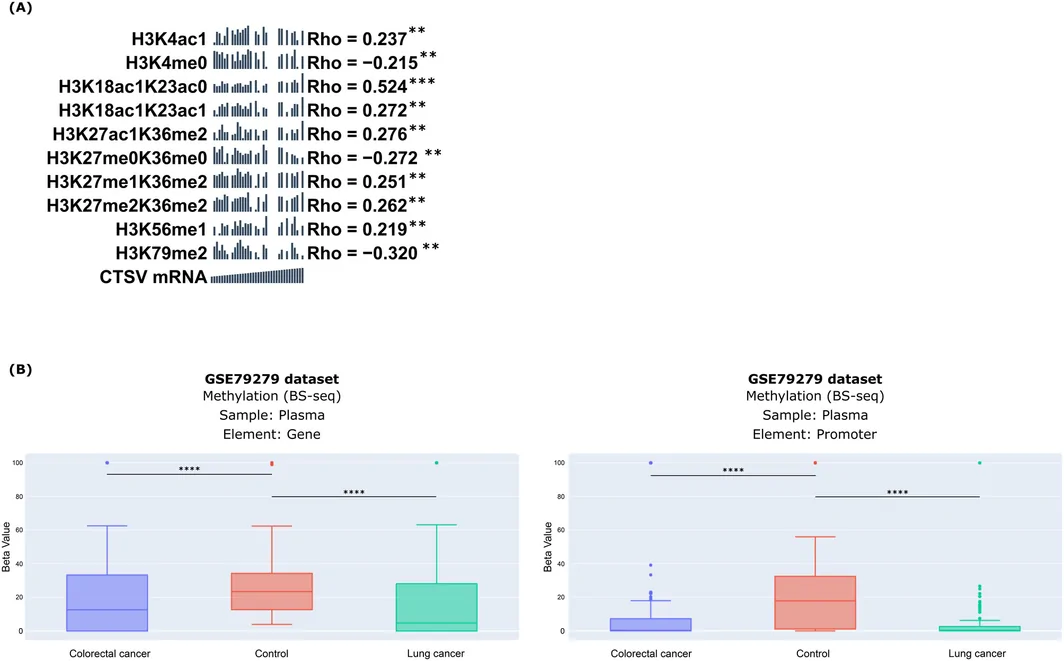
Epigenetic regulation and circulating methylation patterns of *CTSV*. (A) Spearman's correlation between *CTSV* expression and histone H3 modifications quantified by DepMap across 25 human BC cell lines. Right panel: Spearman's correlation coefficient (ρ). *p < 0.05; **p < 0.01; ***p < 0.001. (B) Quantitative estimation of cell‐free *CTSV* DNA methylation in plasma bisulfite sequencing (BS‐seq) samples from healthy individuals and cancer patients (GSE79279 dataset).

Histone profiling (DepMap) revealed 10 histone H3 modifications significantly linked to *CTSV*, with H3K18ac1K23ac0 (an active transcription marker) being the most enriched (ρ = 0.524; *p* < 0.01) (Figure 9A). Together, DNA hypomethylation and histone modifications strongly contribute to *CTSV* overexpression in BC.

### 3.7 *CTSV* Hypomethylation Is Detectable in Cell‐Free DNA (cfDNA)

Building on our exosome findings (Figure 2D), we tested whether *CTSV* hypomethylation signatures could also be detectable in cfDNA. Plasma‐derived cfDNA showed a higher prevalence of hypomethylated *CTSV* CpG sites in patients with colorectal and lung tumors compared with healthy controls (Figure 9B). These results highlight the potential of cfDNA methylation as a noninvasive approach to assess *CTSV* methylation status, offering a promising biomarker for monitoring patients' with BC.

### 3.8 *CTSV* Upregulation Predicts Unique Tumor Microenvironment in BC Patients

**FIGURE 10:**
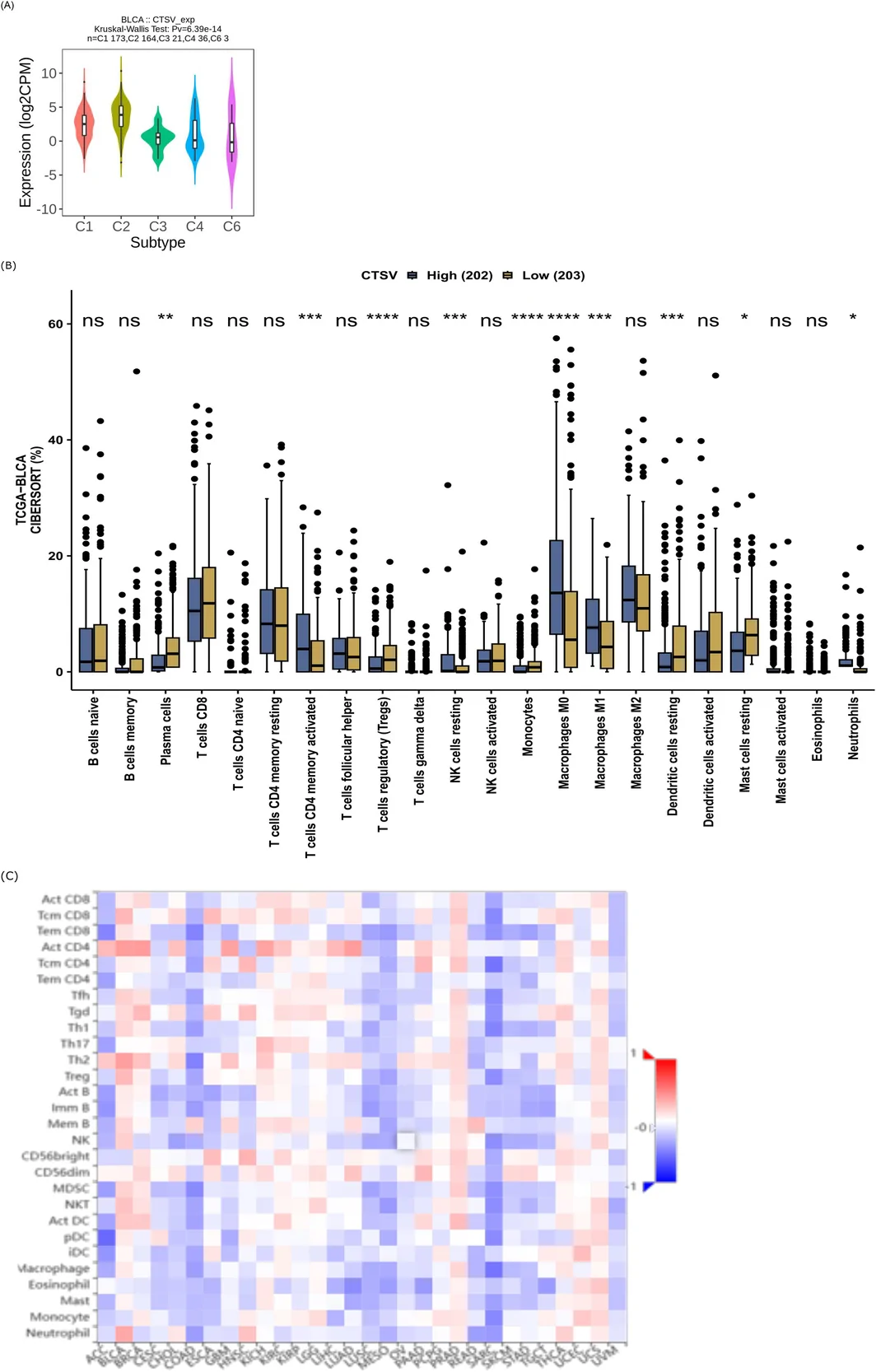
Correlation of *CTSV* expression with the tumor‐infiltrating immune cells in BC patients. (A) *CTSV* expression across immune subtypes in the TCGA‐BLCA samples. C1: Wound‐healing (*n* = 173), C2: INF‐γ–dominant (*n* = 164), C3: Inflammatory (*n* = 21), C4: Lymphocyte‐depleted (*n* = 36), and C6: TGF‐ꞵ dominant (*n* = 3) (*p* = 6.39e‐14 by Kruskal–Wallis test). (B) Relative proportions of 22 infiltrating immune cells in *CTSV* ^*high*^ and *CTSV* ^*low*^ expression groups from the TCGA‐BLCA cohort. The immune cell infiltration scores were estimated using the CIBERSORT algorithm. (C) Heatmap showing the correlation between *CTSV* expression and tumor‐infiltrating immune cells.

Immune subtype analysis (TISIDB) showed that *CTSV* expression was highest in the C2 (INF‐γ dominant) and C1 (wound healing) subtypes (Figure 10A). *CTSV* ^*high*^ tumors exhibited higher stromal fraction, intratumoral heterogeneity, TIL infiltration, macrophage regulation, IFN‐γ, and TGF‐β signaling (Figure S3).

CIBERSORT‐based immune cell deconvolution revealed that *CTSV* ^*high*^ tumors were enriched in activated CD4^+^ memory T cells, resting NK cells, M0/M1 macrophages, and neutrophils, whereas *CTSV* ^*low*^ tumors had more plasma cells and regulatory T cells (Tregs) (Figure 10B). TISIDB findings supported these associations (Figure 10C).

**FIGURE 11:**
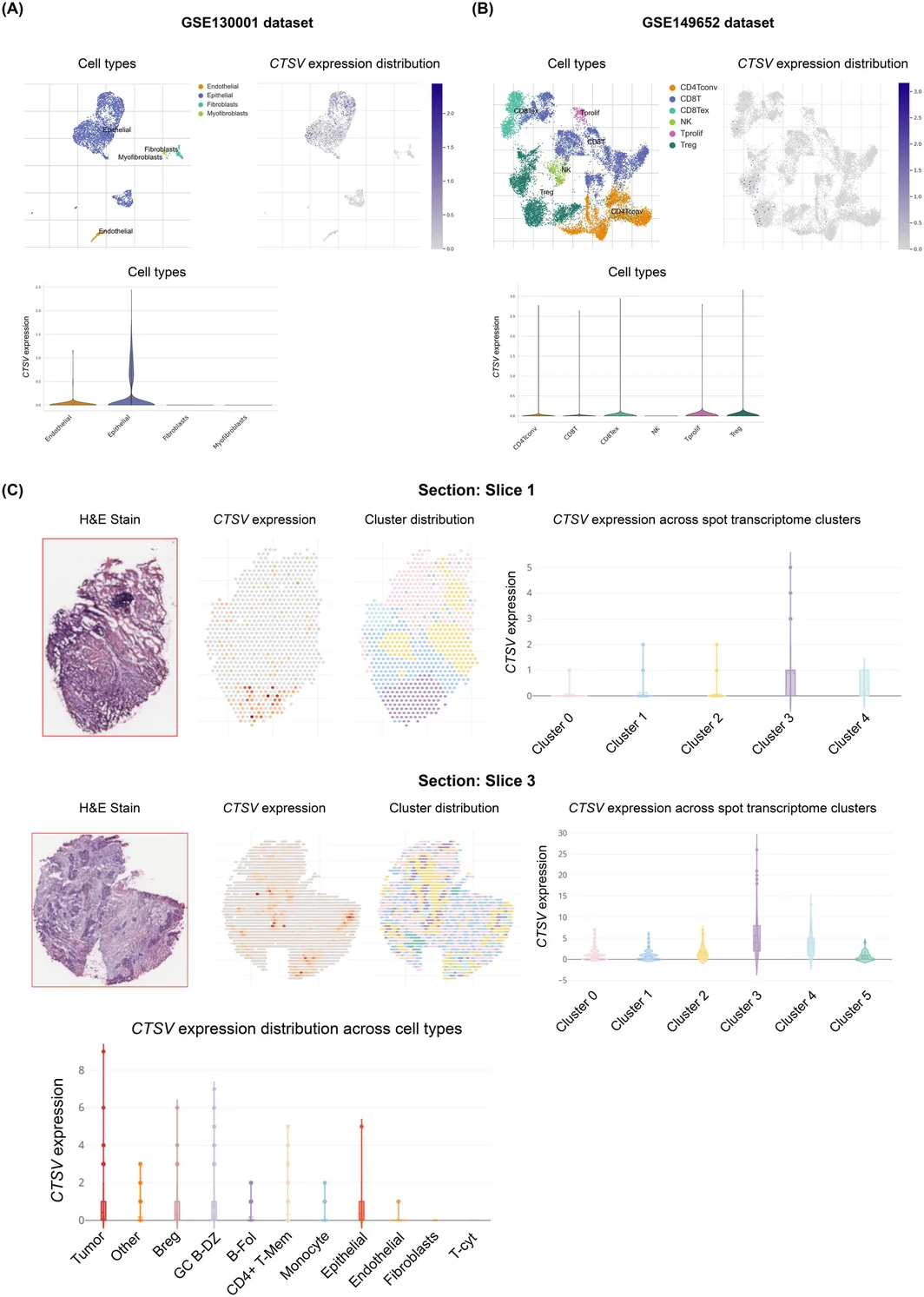
*CTSV* expression across single‐cell and spatial transcriptomics data. (A) UMAP plot of 4129 cells post‐quality control and filtering, grouped by minor cell types. The violin plot illustrates the distribution of *CTSV* expression in different cell types identified in the GSE130001 dataset. (B) UMAP plot of 15 538 cells showing *CTSV* expression in different cell types. The violin plot shows the distribution of *CTSV* expression levels in different cell types identified in the GSE149652 dataset. (C) Upper panel (Slice 1): H&E staining, spatial cluster distribution and *CTSV* expression levels between clusters in fresh frozen tissue from human treatment‐naïve muscle‐invasive BC (GSM5224027 dataset). Number of spots: 768. Number of genes: 13.499 genes; Lower panel (Slice 3): H&E staining, spatial cluster distribution and *CTSV* expression levels between clusters in fresh frozen tissue from human treatment‐naïve muscle‐invasive BC (dataset GSM5224029). Number of spots: 1469. Number of genes: 12.367 genes; Bottom panel: *CTSV* expression distribution across cell types. B‐Fol, follicular B cells; Breg, regulatory B cells; CD4^+^ T‐Mem, CD4^+^ T memory cells; GC B‐DZ, germinal center B cells in the dark zone; T‐cyt, cytotoxic T cells.

The single‐cell analysis showed that *CTSV* was primarily expressed in endothelial and Treg cells (Figure 11A,B), while spatial transcriptome analysis showed increased expression in tumor cells, germinal center B cells in the dark zone (GC B‐DZ), and regulatory B cells (B‐regs) (Figure 11C), which formed distinct cellular niches, most clearly seen in slice 3 (Figure 11C). Collectively, these data support the idea that *CTSV*‐expressing cells help shape and reside in multiple spatially distinct immune‐infiltrated niches.

### 3.9 Potential Therapeutic Drugs Targeting *CTSV*

**FIGURE 12:**
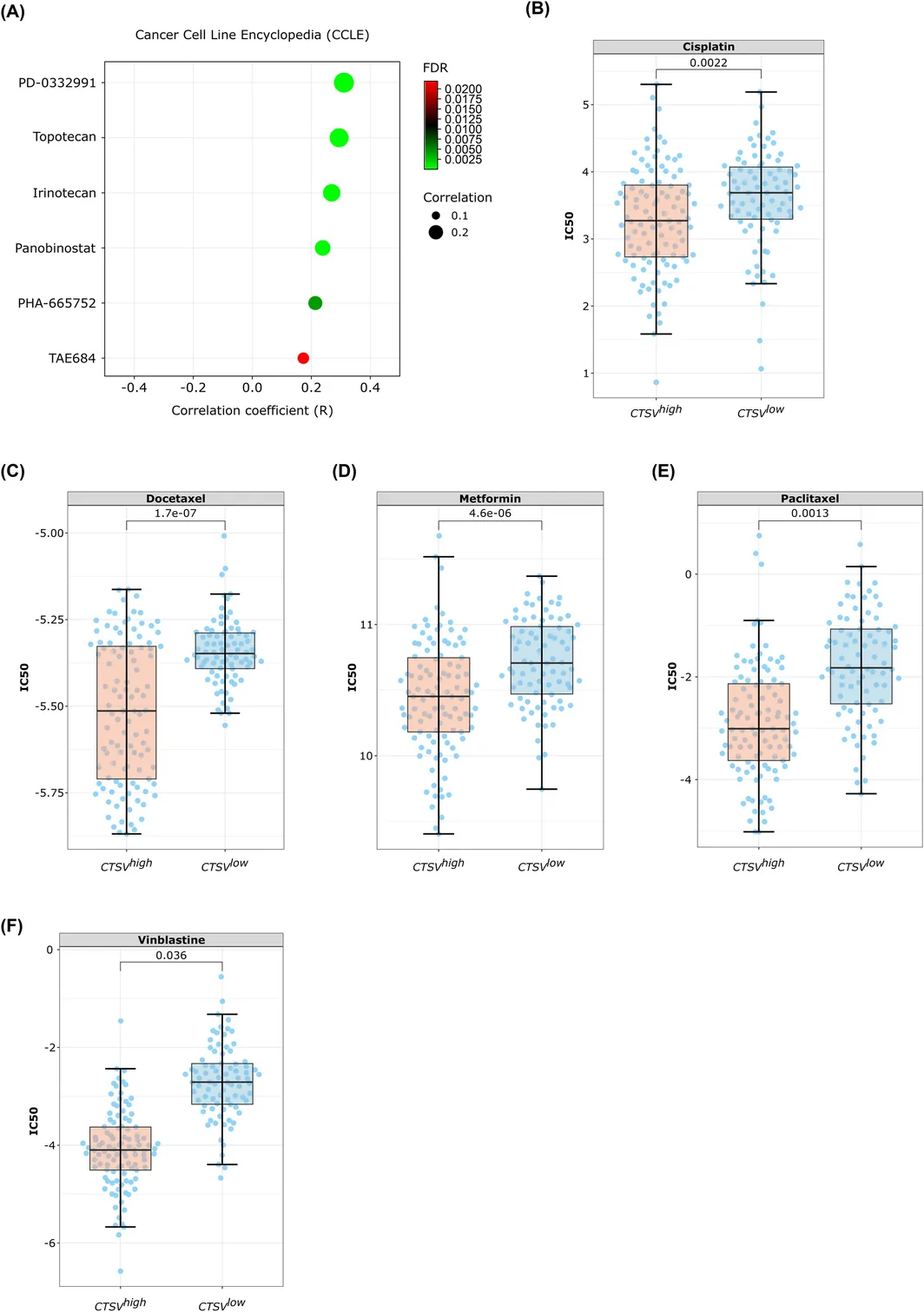
Drug sensitivity of *CTSV*. (A) Correlation between drug sensitivity and mRNA expression level of *CTSV* based on the RNAactDrug database. (B–F) Boxplots showing the difference in the IC50 values for cisplatin (B), docetaxel (C), metformin (D), paclitaxel (E), and vinblastine (F) between the *CTSV* ^*high*^ and *CTSV* ^*low*^ expression groups of TCGA‐BLCA patients. *p*‐values were calculated using the Wilcoxon rank‐sum test.

Drug sensitivity analyses (RNAactDrug) revealed that *CTSV* ^*high*^ cell lines were more responsive to palbociclib, topotecan, irinotecan, panobinostat, PHA‐665752, and TAE684 (Figure 12A). Further CPADS analysis showed that *CTSV* ^*high*^ patients were more sensitive to cisplatin, docetaxel, metformin, paclitaxel, and vinblastine (Figure 12B–F). These findings suggest that *CTSV*‐targeted therapeutic strategies may enhance BC treatment efficacy.

## 4 Discussion

BC stands as the most common malignant tumor of the urinary tract, characterized by high rates of recurrence, progression, and metastasis. Although therapeutic advances, including immune checkpoint inhibitors and antibody‐drug conjugates, have expanded treatment options, these strategies have not substantially improved survival, underscoring the need for novel biomarkers and therapeutic targets.

*CTSV*, a lysosomal cysteine peptidase, regulates processes such as ECM remodeling and immunomodulation [12]. Its overexpression has been reported in several cancers [13, 14], but its epigenetic and post‐transcriptional regulation remains poorly understood. Our findings provide the first evidence of *CTSV*'s epigenetic regulation in BC and reveal its association with clinical outcomes and immune activity.

*CTSV* has been implicated in metastasis through ECM remodeling, immune modulation, histone regulation, and glycosylation‐dependent secretion [4, 12]. Across multiple cohorts, we showed that its expression increased with pathological grade and was associated with higher metastatic potential. Additionally, we found that its expression was strongly influenced by other clinicopathologic and molecular features, resulting in worse outcomes. Survival analyses, logistic regression, and ROC curves confirmed *CTSV* as a robust diagnostic and prognostic biomarker, supporting its value for precision oncology.

Urinary extracellular vesicles (uEVs) are a noninvasive diagnostic tool in BC. Recent proteomic studies have identified upregulated signatures in uEVs from non‐muscle‐invasive BC patients. Given *CTSV*'s role as a circulating biomarker in lung cancer [15], our findings that *CTSV* is enriched in uEVs and other biofluids from various malignancies (e.g., esophageal cancer, GBM, and melanoma) suggest its potential as a circulating biomarker for BC.

We demonstrated that *CTSV* overexpression in BC is associated with DNA hypomethylation at promoter and shore regions, as well as with histone modifications (H3K18ac1K23ac0). Similar hypomethylation patterns have been observed in other diseases [16, 17], while cathepsin L, a homologous protease [12], undergoes methylation‐dependent silencing in B‐cell lymphoma [18], highlighting a conserved mechanism of cathepsin regulation. Furthermore, *CTSV* expression was positively correlated with the methyltransferases *DNMT1* and *DNMT3B*, an association that may reflect a broader context of epigenomic reprogramming in which the maintenance methylation machinery paradoxically favors *CTSV* upregulation, possibly through gene body methylation (gbM)‐mediated transcriptional stabilization [10, 11]. Indeed, DNMT‐dependent gbM is increasingly recognized as a mechanism of oncogene stabilization [10], and the positive correlation between cg14316246 methylation and *CTSV* mRNA expression identified here is consistent with this model. Nevertheless, this association remains correlative, and causal validation through DNMT inhibitor treatment, bisulfite pyrosequencing upon DNMT knockdown, or luciferase reporter assays will be necessary to establish a direct mechanistic link.

The coordinated interplay between the acetylation of H3K18 and H3K23 illustrates the functional synergy between histone modifications, as these marks frequently co‐occur, share regulatory mechanisms, and jointly influence critical processes such as H3K4 methylation and transcriptional activation in cancer [19]. H3K18ac enrichment at promoters correlates with BC progression and helps identify understaged tumors that require cystectomy [20]. H3K23ac, though less studied, promotes oncogenic transcription via *TRIM24* in breast [21] and prostate cancers [22] and contributes to glioblastoma progression through *STAT3* signaling [23]. In BC, *TRIM24* is associated with *NF‐κB* and *AKT* activation [24]. Our analysis demonstrated a correlation between H3K18/23 modifications and *CTSV* expression, supporting a chromatin landscape conducive to *CTSV* upregulation. Further studies are needed to determine whether these epigenetic changes act as early drivers or as secondary events sustaining malignancy.

*CTSV* was strongly associated with the IFN‐γ‐dominant (C2) immune subtype, characterized by high infiltration, cytokine signaling, and T‐cell activation. *CTSV* ^*high*^ tumors showed enrichment in immune‐related pathways, proliferation, wound healing, and altered proportions of macrophages and T cells. Mechanistically, experimental evidence suggests that *CTSV* activates NF‐κB signaling, promoting pro‐inflammatory cytokines (*IL‐1β*, *IL‐6*, *IL‐8*, *TNF‐α*) and sustaining *IL‐17A*‐driven inflammation [25]. It also activates the *NLRP3* inflammasome, amplifying *IL‐1β* release and fostering chronic inflammation that recruits immunosuppressive cells, thereby promoting tumor progression and immune dysfunction [26].

Our single‐cell and spatial analyses revealed *CTSV* expression in endothelial cells, Tregs, germinal center B cells, and tumor cells, creating niches that shape the tumor microenvironment (TME). Although these findings are based on convergent analyses across multiple independent datasets (comprising over 19 000 single cells from nine patients) and are consistent across single‐cell and spatial transcriptomic platforms, protein‐level validation through immunofluorescence or flow cytometry co‐staining is necessary to confirm cell‐type‐specific *CTSV* expression. Endothelial *CTSV* promotes basement membrane remodeling and angiogenesis, similar to cathepsin L's pro‐angiogenic role in breast and gastric cancers [12, 27]. In Tregs/Bregs, *CTSV* may facilitate migration, antigen processing, and immune tolerance [28]. For example, excessive antigen degradation by *CTSV* could generate tolerogenic peptide repertoires, dampening effector T‐cell responses. In Bregs, *CTSV* may support tolerogenic MHC‐II presentation, reinforcing immunosuppression. Although autophagy is indispensable for Treg survival and metabolic homeostasis, and cysteine cathepsins, including *CTSV*, are core executors of lysosomal proteolysis in this pathway, no study has yet directly examined *CTSV* function in Treg cells [29]. Nuclear *CTSV* also regulates histones and the cell cycle [14], potentially enhancing proliferation in germinal center B cells.

Emerging therapeutic insights reinforce *CTSV* as a potential target. Preclinical studies with *CTSV*‐neutralizing antibodies blocked metastasis in lung cancer [13]. Considering its prevalence in high‐grade BC and correlation with chemoresistant phenotypes, therapeutic strategies may involve combining chemotherapy with agents that block *NF‐κB* or enhance the immune response. Current cisplatin‐based combinations (MVAC, GC, PGC) show partial efficacy, while taxanes provide modest benefits in refractory cases [30]. Given *CTSV*'s role in inflammation and immune dysfunction, targeting this protease may advance personalized therapies in BC.

In summary, *CTSV* overexpression in BC is associated with advanced grade, metastasis, poor survival, and immunologically active TMEs. Its regulation involves DNA hypomethylation and histone modifications and may contribute to shaping immune and endothelial niches within tumors. *CTSV* detection in uEVs highlights its promise as a noninvasive biomarker. Future studies should include loss‐ and gain‐of‐function experiments in bladder cancer cell lines (siRNA‐mediated knockdown and stable overexpression) to assess the direct effects of *CTSV* on cell proliferation, migration, invasion, and EMT marker expression (E‐cadherin, N‐cadherin, vimentin). In vivo xenograft models will be critical to confirm tumor growth and metastasis phenotypes. Furthermore, future studies should clarify whether *CTSV*'s epigenetic regulation drives tumor initiation or sustains progression, and whether targeting *CTSV* could enhance therapeutic efficacy.

## Author Contributions

**Edivaldo Herculano Correa de Oliveira:** supervision, funding acquisition, project administration, conceptualization, writing – review and editing, resources. **Fabio Albuquerque Marchi:** data curation, formal analysis, writing – review and editing. **Wallax Augusto Silva Ferreira:** writing – original draft, writing – review and editing, conceptualization, data curation, methodology, formal analysis, visualization, validation, investigation, funding acquisition, software, resources. **Ana Paula Drummond Rodrigues:** data curation, writing – review and editing. **Rolando André Rios Villacis:** formal analysis, writing – review and editing. **Glauco Akelinghton Freire Vitiello:** data curation, writing – review and editing.

## Funding

This work was funded by the Coordination for the Improvement of Higher Education Personnel (CAPES—Brazil) (Process n° 88882.445289/2019–01 and 88887.799750/2022–00), Conselho Nacional de Desenvolvimento Científico e Tecnológico (CNPq) (Process n° 153 210/2024–9), and Evandro Chagas Institute (IEC/SVSA/MS—Brazil).

## Consent

The authors have nothing to report.

## Conflicts of Interest

The authors declare no conflicts of interest.

## Supporting information


**Figure S1:** DNA methylation patterns within the *CTSV* (cathepsin V) gene in several human bladder cancer cell lines from the DepMap database. The y‐axis: represents 23 different human bladder cancer cell lines; x‐axis: shows specific genomic coordinates on chromosome 9 corresponding to individual CpG sites within *CTSV*. The color gradient from blue to red represents methylation beta values from 0.0 (nonmethylated) to 1.0 (fully methylated). Bubble size scale: (i) smallest bubbles: 5 reads coverage; (ii) intermediate sizes, 10, 15 and 20 reads coverage; largest bubbles: 25+ reads coverage.

**Figure S2:** Dynamics of DNA methylation across CpG sites of *CTSV* in TCGA‐BLCA patients. Heatmap showing the methylation levels of *CTSV* between different CpGs sites (probes) integrating ethnicity, race, age, vital status and genomic regions of CpG sites from TCGA‐BLCA.

**Figure S3:** Differential expression analysis of the *CTSV* gene between high (*n* = 202) and low groups (*n* = 203) in the TCGA‐BLCA cohort according to different immune score parameters. ****p < 0.0001, ***p < 0.001, **p < 0.01, *p < 0.05, ns: not significant.

**Table S1:** Association of *CTSV* expression with clinicopathological characteristics in BC patients from TCGA‐BLCA cohort, GSE32894, GSE31684, GSE87304, GSE169455, GSE48075, GSE93527. Bold numbers indicate that they were statistically significant (*p*‐value < 0.05).

## Data Availability

The data that support the findings of this study are available in SORC at http://bio‐bigdata.hrbmu.edu.cn/SORC/index.jsp. These data were derived from the following resources available in the public domain: exoRBase 2.0 database, https://doi.org/10.1093/nar/gkab1085/TIMER2.0, http://timer.cistrome.org/GEPIA2, http://gepia2.cancer/pku.cn/GEO2R, https://www.ncbi.nlm.nih.gov/geo/geo2r/Human‐Protein‐Atlas‐(HPA), https://www.proteinatlas.org/DepMap, https://depmap.org/portal/R2‐platform, https://hgserver1.amc.nl/cgi/bin/r2/main.cgi/Kaplan‐Meier‐plotter, https://kmplot.com/analysis/PROGgeneV2, http://genomics.jefferson.edu/proggene/index.php/cBioportal, https://www.cbioportal.org/GSCA‐database, http://bioinfo.life.hust.edu.cn/GSCA/CAMOIP, https://www.camoip.net/UALCAN, https://ualcan.path.uab.edu/Methsurv, https://biit.cs.ut.ee/methsurv/SMART, http://www.bioinfo/zs.com/smartapp/IMOPAC, https://www.hbpding.com/IMOPAC/cfOmics‐database, https://cfomics.ncRNAlab.org/TISIDB‐database, http://cis.hku.hk/TISIDB/TISCH2‐database, http://tisch.comp/genomics.org/home/SORC‐database, http://bio‐bigdata.hrbmu.edu.cn/SORC/index.jsp.

## References

[1] L. M. van Hoogstraten , A. Vrieling , A. G. van der Heijden , M. Kogevinas , A. Richters , and K. LAJNrCo , “Global Trends in the Epidemiology of Bladder Cancer: Challenges for Public Health and Clinical Practice,” Nature Reviews. Clinical Oncology 20, no. 5 (2023): 287–304. 10.1038/s41571-023-00744-3 36914746

[2] S. V. Lindskrog , F. Prip , P. Lamy , et al., “An Integrated Multi‐Omics Analysis Identifies Prognostic Molecular Subtypes of Non‐Muscle‐Invasive Bladder Cancer,” 12, no. 1 (2021): 2301. 10.1038/s41467-021-22465-w PMC8052448 33863885

[3] W. Ma , C. Li , Y. Liu , et al., “Multi‐Omics Analysis Identifies the Heterogeneity and Prognosis in Bladder Cancer,” (2021).

[4] J. I. Warrick , H. Al‐Ahmadie , D. M. Berman , et al., “International Society of Urological Pathology Consensus Conference on Current Issues in Bladder Cancer. Working Group 4: Molecular Subtypes of Bladder Cancer—Principles of Classification and Emerging Clinical Utility,” American Journal of Surgical Pathology 48, no. 1 (2024): e32–e42. 37199442 10.1097/PAS.0000000000002053

[5] T. Li , J. Fu , Z. Zeng , et al., “TIMER2.0 for Analysis of Tumor‐Infiltrating Immune Cells,” Nucleic Acids Research 48, no. W1 (2020): W509–W514. 32442275 10.1093/nar/gkaa407 PMC7319575

[6] Z. Tang , C. Li , B. Kang , G. Gao , C. Li , and Z. ZJNar , “GEPIA: A Web Server for Cancer and Normal Gene Expression Profiling and Interactive Analyses,” Nucleic Acids Research 45, no. W1 (2017): W98–W102. 28407145 10.1093/nar/gkx247 PMC5570223

[7] L. Mengual , M. Burset , E. Ars , et al., “DNA Microarray Expression Profiling of Bladder Cancer Allows Identification of Noninvasive Diagnostic Markers,” Journal of Urology 182, no. 2 (2009): 741–748. 19539325 10.1016/j.juro.2009.03.084

[8] DepMap BJDQP , “Current DepMap Release Data, Including CRISPR Screens, PRISM Drug Screens, Copy Number, Mutation, Expression, and Fusions,” (2024).

[9] Y. Han , Y. Wang , X. Dong , et al., “TISCH2: Expanded Datasets and New Tools for Single‐Cell Transcriptome Analyses of the Tumor Microenvironment,” Nucleic Acids Research 51, no. D1 (2023): D1425–D1431. 36321662 10.1093/nar/gkac959 PMC9825603

[10] X. Yang , H. Han , D. D. De Carvalho , F. D. Lay , P. A. Jones , and L. GJCc , “Gene Body Methylation Can Alter Gene Expression and Is a Therapeutic Target in Cancer,” Cancer Cell 26, no. 4 (2014): 577–590. 25263941 10.1016/j.ccr.2014.07.028 PMC4224113

[11] M. H. McGuire , S. K. Dasari , H. Yao , et al., “Gene Body Methylation of the Lymphocyte‐Specific Gene CARD11 Results in Its Overexpression and Regulates Cancer mTOR Signaling,” Molecular Cancer Research 19, no. 11 (2021): 1917–1928. 34348992 10.1158/1541-7786.MCR-20-0753 PMC8568653

[12] F. Lecaille , T. Chazeirat , A. Saidi , and G. Lalmanach , “Cathepsin V: Molecular Characteristics and Significance in Health and Disease,” Molecular Aspects of Medicine 88 (2022): 101086. 35305807 10.1016/j.mam.2022.101086

[13] L. Zhu , Q. Zeng , J. Wang , F. Deng , and S. Jin , “Cathepsin V Drives Lung Cancer Progression by Shaping the Immunosuppressive Environment and Adhesion Molecules Cleavage,” Aging 15, no. 23 (2023): 13961–13979. 38078882 10.18632/aging.205278 PMC10756122

[14] N. Sereesongsaeng , J. F. Burrows , C. J. Scott , K. Brix , and R. E. Burden , “Cathepsin V Regulates Cell Cycle Progression and Histone Stability in the Nucleus of Breast Cancer Cells,” Frontiers in Pharmacology 14 (2023): 1271435. 38026973 10.3389/fphar.2023.1271435 PMC10657903

[15] L. Yang , Q. Zeng , Y. Deng , Y. Qiu , W. Yao , and Y. Liao , “Glycosylated Cathepsin V Serves as a Prognostic Marker in Lung Cancer,” Frontiers in Oncology 12 (2022): 876245. 35494076 10.3389/fonc.2022.876245 PMC9043764

[16] Y. P. Leng , Y. S. Ma , X. G. Li , et al., “L‐Homocysteine‐Induced Cathepsin V Mediates the Vascular Endothelial Inflammation in Hyperhomocysteinaemia,” British Journal of Pharmacology 175, no. 8 (2018): 1157–1172. 28631302 10.1111/bph.13920 PMC5866977

[17] R. O. Bahado‐Singh , S. Vishweswaraiah , B. Aydas , et al., “Artificial Intelligence and Leukocyte Epigenomics: Evaluation and Prediction of Late‐Onset Alzheimer's Disease,” PLoS One 16, no. 3 (2021): e0248375. 33788842 10.1371/journal.pone.0248375 PMC8011726

[18] D. Jean , N. Rousselet , and R. Frade , “Expression of Cathepsin L in Human Tumor Cells Is Under the Control of Distinct Regulatory Mechanisms,” Oncogene 25, no. 10 (2006): 1474–1484. 16261157 10.1038/sj.onc.1209196

[19] G. C. Fox , K. F. Poncha , B. R. Smith , et al., “H3K18 & H3K23 Acetylation Directs Establishment of MLL‐Mediated H3K4 Methylation,” bioRxiv: The Preprint Server for Biology 300 (2024): 107527. 10.1016/j.jbc.2024.107527 PMC11338103 38960040

[20] J. Ellinger , A. C. Schneider , A. Bachmann , G. Kristiansen , S. C. Muller , and S. Rogenhofer , “Evaluation of Global Histone Acetylation Levels in Bladder Cancer Patients,” Anticancer Research 36, no. 8 (2016): 3961–3964. 27466500

[21] L. Ma , L. Yuan , J. An , M. C. Barton , Q. Zhang , and Z. Liu , “Histone H3 Lysine 23 Acetylation Is Associated With Oncogene TRIM24 Expression and a Poor Prognosis in Breast Cancer,” Tumour Biology: The Journal of the International Society for Oncodevelopmental Biology and Medicine 37, no. 11 (2016): 14803–14812. 27638829 10.1007/s13277-016-5344-z

[22] A. C. Groner , L. Cato , J. de Tribolet‐Hardy , et al., “TRIM24 Is an Oncogenic Transcriptional Activator in Prostate Cancer,” Cancer Cell 29, no. 6 (2016): 846–858. 27238081 10.1016/j.ccell.2016.04.012 PMC5124371

[23] D. Lv , Y. Li , W. Zhang , et al., “TRIM24 Is an Oncogenic Transcriptional Co‐Activator of STAT3 in Glioblastoma,” Nature Communications 8, no. 1 (2017): 1454. 10.1038/s41467-017-01731-w PMC5682287 29129908

[24] D. Xue , X. Zhang , X. Zhang , et al., “Clinical Significance and Biological Roles of TRIM24 in Human Bladder Carcinoma,” Tumour Biology: The Journal of the International Society for Oncodevelopmental Biology and Medicine 36, no. 9 (2015): 6849–6855. 25846736 10.1007/s13277-015-3393-3

[25] Y. Xia , M. Ge , L. Xia , G. Shan , and H. Qian , “CTSV (Cathepsin V) Promotes Bladder Cancer Progression by Increasing NF‐kappaB Activity,” Bioengineered 13, no. 4 (2022): 10180–10190. 35443863 10.1080/21655979.2022.2061278 PMC9162008

[26] Y. Leng , R. Chen , R. Chen , et al., “HMGB1 Mediates Homocysteine‐Induced Endothelial Cells Pyroptosis via Cathepsin V‐Dependent Pathway,” Biochemical and Biophysical Research Communications 532, no. 4 (2020): 640–646. 32912629 10.1016/j.bbrc.2020.08.091

[27] M. O. Platt and W. A. Shockey , “Endothelial Cells and Cathepsins: Biochemical and Biomechanical Regulation,” Biochimie 122 (2016): 314–323. 26458976 10.1016/j.biochi.2015.10.010 PMC4747805

[28] D. Lv , Y. Fei , H. Chen , et al., “Crosstalk Between T Lymphocyte and Extracellular Matrix in Tumor Microenvironment,” Frontiers in Immunology 15 (2024): 1340702. 38690275 10.3389/fimmu.2024.1340702 PMC11058664

[29] V. Kaminskyy and Zhivotovsky BJBeBA‐P, Proteomics , “Proteases in Autophagy,” Biochimica et Biophysica Acta 1824, no. 1 (2012): 44–50. 21640203 10.1016/j.bbapap.2011.05.013

[30] V. Goel , A. Jain , D. A. Basu , et al., “A Comparative Study of Gemcitabine‐Cisplatin vs. Dose‐Dense MVAC (Methotrexate, Vinblastine, Doxorubicin, and Cisplatin) as Neoadjuvant Chemotherapy for Muscle‐Invasive Bladder Cancer: A Single‐Institution Experience,” Cureus 17, no. 5 (2025): e85071. 40585605 10.7759/cureus.85071 PMC12206345

