# Supplementary material for: Cathepsin V ( CTSV ) Shapes the Epigenetic–Immune Landscape of Bladder Cancer and Emerges as a Noninvasive Biomarker
Source: Int J Urol. 2026 Aug 26;33(9):e70609. doi: 10.1111/iju.70609 (PMC13519135; doi:10.1111/iju.70609)

Supplementary Figure 2

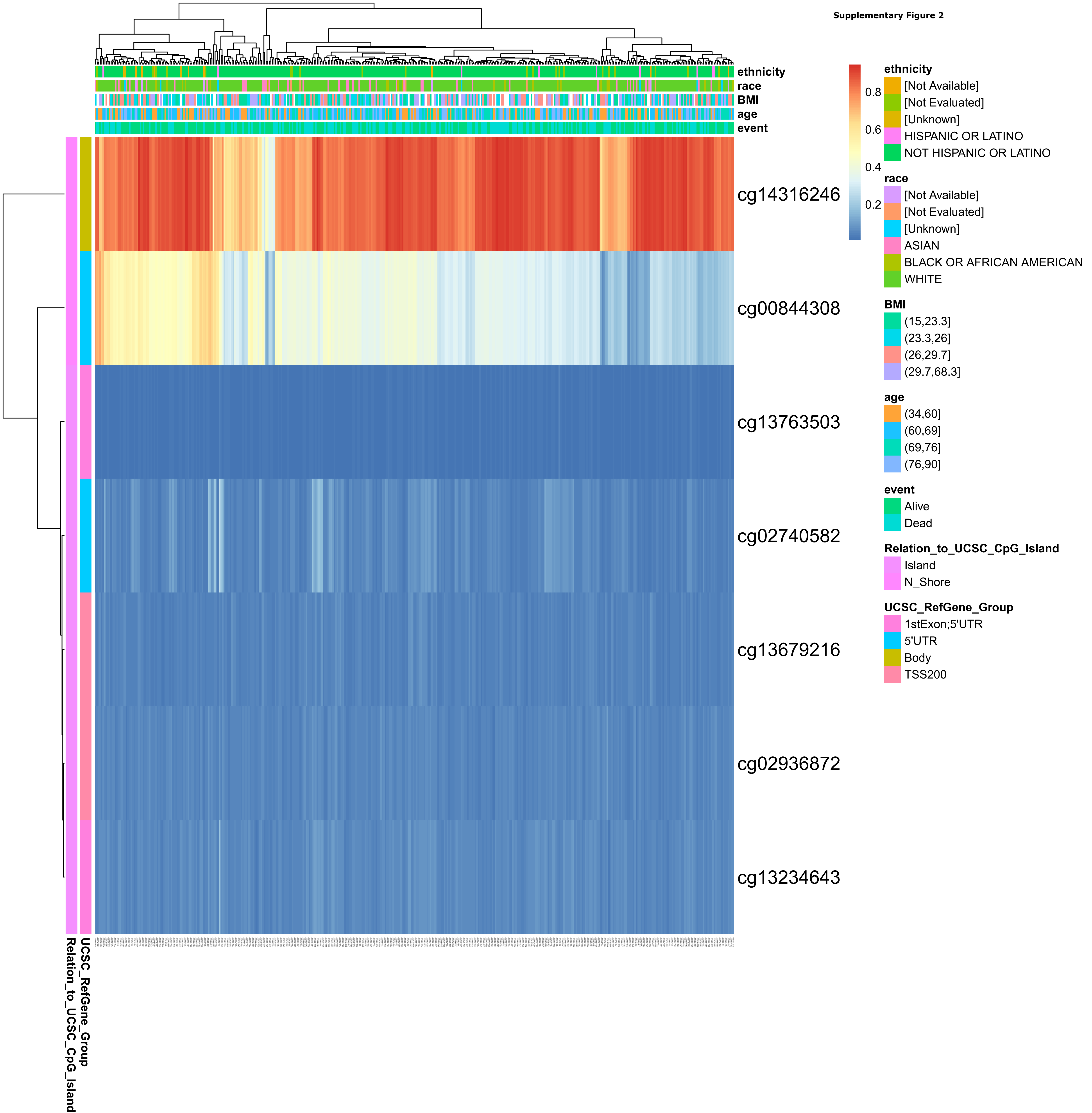

Supplement: Supplementary file 1 [file IJU-33-0-s001.pdf]
