# Supplementary material for: Cathepsin V ( CTSV ) Shapes the Epigenetic–Immune Landscape of Bladder Cancer and Emerges as a Noninvasive Biomarker
Source: Int J Urol. 2026 Aug 26;33(9):e70609. doi: 10.1111/iju.70609 (PMC13519135; doi:10.1111/iju.70609)
Supplement: Supplementary file 2 [file IJU-33-0-s002.pdf]

Supplementary Figure 3

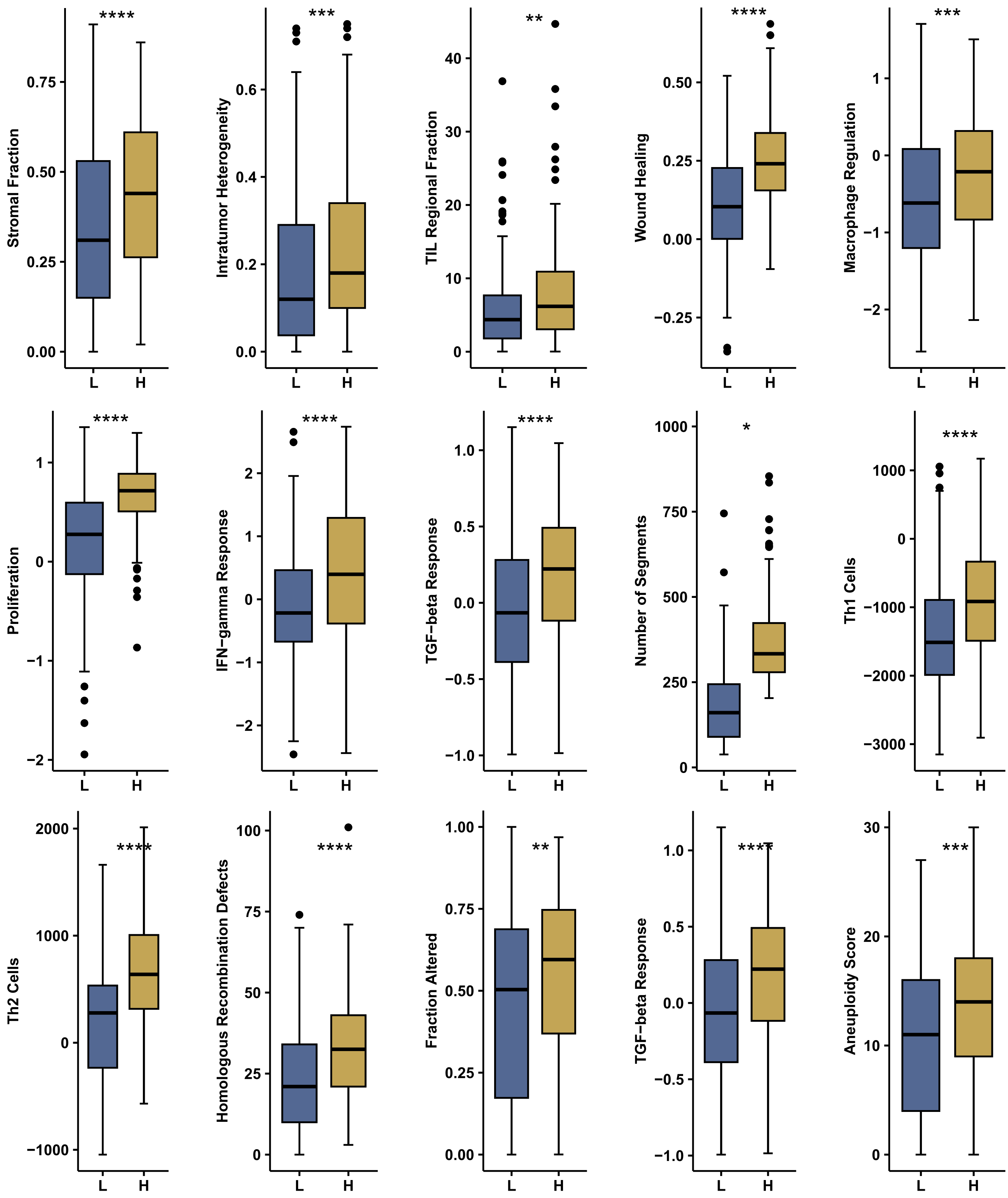

TCGA−Cohort:
BLCA

CTSV:
1) H: High (n = 202)
2) L: Low (n = 203)

Wilcoxon test:
******P<0.0001**
*****P<0.001**
****P<0.01**
***P<0.05**
ns:not significant
